# Somatosensory amplification and fear of cancer recurrence: Moderating effects of emotion regulation difficulties

**DOI:** 10.1007/s00520-026-10914-5

**Published:** 2026-06-22

**Authors:** Shimrit Daches, Ohad Zohar, Shahaf Bitan, Ilanit Hasson-Ohayon

**Affiliations:** Department of Psychology, Bar-Ilan University, Ramat-Gan, Israel

**Keywords:** Cancer, Emotion Regulation Difficulties, Fear of Cancer Recurrence, Somatosensory Amplification, Survivors

## Abstract

**Purpose:**

Fear of cancer recurrence (FCR) is one of the most prevalent and distressing psychological concerns among cancer survivors. This study examined the association between somatosensory amplification (SSA) and FCR and tested whether emotion regulation (ER) difficulties moderate this relationship.

**Methods:**

A sample of 116 adult cancer survivors (*M*age = 47.24) completed validated self-report measures assessing SSA, ER difficulties, and FCR. Hierarchical regression analyses were conducted, controlling for anxiety symptom severity, age, gender, and time since treatment completion. Results: Neither SSA nor ER difficulties were associated with FCR. Yet, the interaction between SSA and ER difficulties was statistically significant. Specifically, the positive association between SSA and FCR was evident at low and mean levels of ER difficulties, but not at high levels.

**Conclusions:**

Findings suggest that the relationship between heightened bodily sensitivity and FCR may differ according to levels of ER difficulties. Future research should explore longitudinal pathways and relevant intervention-based outcomes.

**Implications for cancer survivors:**

Screening for SSA and ER difficulties in survivorship care may help identify individuals for whom heightened bodily sensitivity is associated with increased FCR.

## Background

Recent advances in cancer care have significantly increased survivorship, with the U.S. survivor population projected to reach 26 million by 2040 (National Cancer Institute). Fear of Cancer Recurrence (FCR) is among the most common psychological concerns reported by survivors and has been identified as a key area for mental health intervention [1]. In response, research on FCR—its predictors and outcomes—has grown in recent years [2].

FCR refers to concerns about cancer returning, progressing, or developing metastases [3]. It is considered an almost universal experience among cancer survivors [4]. A systematic review of 15 studies by Koch et al. [5] found that most survivors experience FCR and that these fears tend to remain stable over time. In a more recent meta-analysis that included data from 9311 respondents worldwide, FCR was found to be prevalent among more than half of survivors, across cancer types and continents, and for all time periods since cancer diagnosis [6]. While a certain level of FCR may be expected and even adaptive, encouraging medical follow-up and health-promoting behaviors, excessive FCR can significantly impair quality of life. A recent meta-analysis by Zhang et al. [2] reported FCR to be positively associated with anxiety, depression, and distress, while negatively associated with optimism, quality of life, and well-being. Moreover, individuals with higher levels of FCR are more likely to meet diagnostic criteria for psychiatric disorders [7]. Notably, the impact of FCR can persist for even a decade after diagnosis [8].

Several theories have been developed to explain FCR, including the cognitive processing model [9] and the self-regulation model of illness [10]. Simonelli et al. [11] highlight that an overlapping component of these theoretical models is that cues, whether internal (e.g., bodily sensations) or external (e.g., social media), trigger FCR cognitive schemas. For example, bodily triggers are interpreted as signs of recurrence, that may activate intrusive thoughts and emotional reactions of fear and distress. FCR persists by increased vigilance to bodily cues and catastrophic interpretations of them [9].

Attention to bodily sensations plays a central role in FCR, as physical sensations are often interpreted as potential indicators of disease return. Crist and Grunfeld [3] noted that ongoing treatment-related side effects or physical symptoms, especially pain, are associated with higher FCR levels [12]. Relatedly, *Somatosensory Amplification* (SSA), defined as the tendency to perceive benign bodily sensations as intense and symptomatic of illness [13], has been linked to health-related fears, including disease phobia [14]. Although the limited number of studies assessing SSA among cancer survivors have not found significant differences in overall SSA levels compared with healthy controls, higher SSA within survivor samples has been associated with greater symptom burden [15, 16]. Specifically, survivors reporting higher SSA also report more severe physical symptoms [15], and among breast cancer survivors, elevated SSA has been associated with higher levels of anxiety, fatigue, depression, and confusion [16].

While SSA and FCR share some features, they are conceptually distinct. SSA is a trait reflecting bodily attentiveness and interpretation, whereas FCR is a cognitive-emotional response focused on concerns about cancer recurrence. SSA may amplify bodily sensations, thereby increasing the likelihood of FCR, but it does not inherently involve recurrence-specific fears. To the best of our knowledge, only two studies examined associations between FCR and variables related to the experience of bodily symptoms. Cunningham et al. [17] found that worry about bodily symptoms by survivors of childhood cancer, aged 8 to 25, was more strongly associated with FCR than the frequency of those symptoms themselves, even after controlling for treatment factors and general anxiety. In contrast, Cessna Palas et al. [18] found no significant association between misinterpretation of bodily symptoms and FCR in adult survivors of colorectal cancer. Notably, misinterpretation in that study was assessed using the hypochondriacal absorption subscale of the MIHT [19], which captures attentional focus on bodily sensations but does not assess whether such sensations are appraised as threatening or distressing. Taken together, not only have very few studies investigated constructs similar to SSA in relation to FCR, but existing results have also been inconsistent, potentially due to differences in methodology or sample characteristics.

It is possible that the association between SSA and FCR is not straightforward, but instead is moderated by an individual’s ability to manage distressing bodily sensations. One key factor that may influence this relationship is emotion regulation (ER), a critical personal resource that shapes how individuals cope with stress, anxiety, and related psychological challenges [20]. ER refers to the processes by which emotional experiences are evaluated, monitored, and modified over time, and plays a central role in determining which emotions we experience, as well as when and how we express them [21, 22].

Literature on ER distinguishes between two complementary perspectives. One body of work focuses on individuals' tendency to engage in specific ER strategies, such as acceptance, suppression, rumination, distraction, and reappraisal, and examines their relative effectiveness in modulating emotional responses [e.g., 23]. This strategy-based approach conceptualizes ER largely as the sum of discrete techniques for managing emotions. However, evidence from the cancer survivorship literature highlights limitations of this perspective. Specifically, a recent meta-analysis reported inconsistent associations (negative, positive, or null) between individual ER strategies and psychological distress [24].

In contrast, other researchers emphasize a broader, multidimensional construct of ER that extends beyond the mere use of specific strategies. This perspective, that is used in the current study, exemplified by Gratz and Roemer’s model, encompasses emotional awareness, understanding, acceptance, impulse control, goal-directed behavior under distress, and the flexible use of context-appropriate strategies [25]. From this viewpoint, ER difficulties reflect broader impairments in emotional awareness, clarity, acceptance, and regulatory flexibility, rather than the mere presence or absence of specific strategies. Converging evidence from multiple empirical studies indicates that ER difficulties are associated with various psychiatric conditions, including anxiety disorders, eating disorders, depression, posttraumatic stress disorder, and substance use disorders [26–28]. Moreover, findings from a healthy adult sample demonstrated that ER difficulties uniquely predict health anxiety beyond the contribution of specific ER strategies [29]. Recent work has also linked ER difficulties to FCR among survivors shortly after treatment completion [30]. Accordingly, the present study focuses on ER difficulties, as this construct captures maladaptive patterns of emotional responding, such as reduced awareness and limited regulatory flexibility, that are particularly relevant to vulnerability for distress and psychopathology [26–28].

Studies across diverse contexts indicate that individual differences in ER difficulties are associated with variability in the strength of associations between psychological symptoms and emotional or somatic outcomes [e.g., 31–33]. For example, Selvi and Bojo [31] reported that ER difficulties intensified the association between perceived stress and the severity of somatic symptoms. ER difficulties have also been shown to moderate the link between perceived stress and well-being indicators, such as life satisfaction and happiness, with stronger negative associations observed among individuals reporting more ER difficulties [32].

In the context of cancer survivorship, related but conceptually distinct ER-related capacities have also been implicated. Hou et al. [33] found that the association between physical symptoms and depressive symptoms was stronger among individuals with lower capacity to maintain positive affect. Complementing this work, Harel et al. [34] demonstrated that inflammatory cytokines were more strongly associated with depressive symptoms among survivors reporting higher emotional awareness, a construct reflecting heightened attention to and differentiation of internal emotional states rather than ER difficulties per se. Together, these findings suggest that individual differences in the processing and regulation of internal experiences may shape the extent to which bodily or biological signals are associated with emotional distress.

Collectively, these findings support the plausibility that ER difficulties function as an individual difference factor that may be related to the strength of the association between bodily threat sensitivity and FCR. Although some cancer survivorship studies have examined related but distinct constructs, such as emotional awareness [34] or the capacity to maintain positive affect [33], the broader literature specifically examining ER difficulties generally suggests that greater ER difficulties are associated with stronger links between stress-related experiences and emotional or somatic distress [e.g., 31–33]. Specifically, heightened sensitivity to bodily sensations may be more closely linked with FCR among individuals who report greater ER difficulties when responding to distressing internal experiences. Accordingly, the present study examined the interaction between SSA and ER difficulties in predicting FCR among cancer survivors in remission. Identifying such an interaction may help clarify how dispositional vulnerabilities are jointly associated with FCR.

As noted above, FCR is one of the most common and distressing psychological concerns among cancer survivors [1]. In the present study, survivorship is defined as the period following completion of active cancer treatment and within 10 years of diagnosis, consistent with prior FCR research [35]. Understanding the factors associated with elevated FCR is therefore essential for explaining why some individuals remain particularly vulnerable to persistent fear during survivorship and for improving psychosocial care. The present study aims to extend and refine theoretical models of FCR [9, 10] by integrating ER difficulties as a moderating factor in the association between SSA and FCR. We hypothesized that both SSA and ER difficulties would be positively associated with self-reported FCR. In addition, we expected ER difficulties to moderate the association between SSA and FCR, such that heightened SSA would be positively associated with FCR among individuals reporting high levels of ER difficulties.

## Methods

### Participants

A total of 119 Hebrew-speaking cancer survivors of various cancer types who were above 18 years of age (30 males, *M* age = 47, *SD* = 13.51) were recruited. Participants were primarily recruited through designated social media (67%) and WhatsApp groups (11%), as well as a mental health rehabilitation clinic at Sheba Medical Center, Israel (22%). In order to be included in the study, participants had to have a cancer diagnosis within the past 10 years, be 18 years or older at the time of diagnosis, have completed active treatment (e.g., intravenous chemotherapy, radiation therapy, surgery) at any time before participation, and have the ability to complete an online survey in Hebrew. Survivors who experienced cancer recurrence were instructed to answer the questionnaires referring to the last episode of acute treatments. 2 participants were excluded due to not meeting the inclusion criteria: one reporting an initial diagnosis before age 18, and another who reported that more than 10 years had passed from the initial diagnosis. One participant failed to complete the self-report scales. Thus, the final sample for further analysis included 116 participants.

An a priori power analysis was conducted using G*Power version 3.1.9.6 to determine the minimum sample size required to test the hypothesized moderation models [36]. Results indicated that the required sample size needed to achieve 80% power for detecting an expected small to medium effect size, at a significance criterion of α = 0.05, was *N* = 113.

### Procedure

The current study was part of a large-scale project on the psychological aspects of cancer recovery [37, 38], approved by both the Ethics Committee at Sheba Medical Center (Approval number: SMC-9838–22) and the Bar-Ilan University Ethics Committee (Approval number: 2023/48), and was carried out in accordance with the Declaration of Helsinki. Research assistants contacted individuals who had indicated interest in the study by phone and provided them with a detailed explanation of the study's purpose. During the call, participants confirmed that they met the inclusion criteria. Participants who met these criteria gave informed consent and were subsequently sent a link to the online survey. Response rate was excellent (96%). The anonymous questionnaires, which took approximately 30 min to complete, were filled out by the participants, who were then thanked and compensated for their time with 70 NIS (approximately $20 USD).

### Measures

The Fear of Cancer Recurrence Inventory – Short Form (FCRI-SF) was developed and validated by Simard and Savard [7] to assess FCR in patients suffering from any type of cancer. The FCRI-SF is a 9-item self-report questionnaire (e.g., 'I am worried or anxious about the possibility of cancer recurrence') that includes a 5-point Likert scale for participants to indicate the degree to which each item describes them, ranging from 0 (= *not at all*) to 4 (= *a great deal*). The nine items are summed to yield a total score ranging from 0 to 36, with higher scores indicating greater FCR. The FCRI-SF demonstrates convergent validity with other FCR measures [7]. In the current study, we used a validated Hebrew version of the FCRI-SF [39]. Internal consistency in the current study was good (Cronbach's α = 0.85).

The Somatosensory Amplification Scale (SSAS; [13] is a 10-item self-report questionnaire assessing the tendency to experience ordinary bodily and visceral sensations as intense, noxious, and disturbing (e.g., 'I can’t stand smoke, smog, or pollutants in the air'). Items are rated on a 5-point Likert scale, where participants indicate the degree to which they are bothered by different somatic and visceral sensations, ranging from 1 (= *not at all true*) to 5 (= *extremely true*). The 10 items are averaged to yield a mean score ranging from 1 to 5, with higher scores indicating greater SSA. The SSAS was found to have adequate internal consistency and test–retest reliability [13]. The questionnaire was translated from English to Hebrew by a bilingual expert, and then independently back-translated into English by another bilingual translator to ensure accuracy and conceptual equivalence. Discrepancies were discussed and resolved by consensus. Internal consistency in the current study was acceptable (Cronbach's α = 0.74).

The Difficulties in Emotion Regulation Scale (DERS; [25]) was developed in order to assess multiple facets of emotion dysregulation. The DERS contains 36 items that are scored on a 5-point Likert scale from 1 (= *almost never*) to 5 (= *almost always*), which indicates the severity of the behavior described in each item. The scale includes six subscales: (1) Nonacceptance of emotional responses (e.g., 'When I’m upset, I become angry with myself for feeling that way'), (2) Difficulties engaging in goal-directed behavior (e.g., 'When I’m upset, I have difficulty getting work done'), (3) Impulse control difficulties (e.g., 'I experience my emotions as overwhelming and out of control'), (4) Lack of emotional awareness (e.g., 'I pay attention to how I feel'), (5) Limited access to emotion regulation strategies (e.g., 'When I’m upset, I believe that I will remain that way for a long time'), and (6) Lack of emotional clarity (e.g., 'I have no idea how I am feeling'). The 36 items are summed to yield a total score ranging from 36 to 180, with higher scores indicating greater difficulties in regulating emotions. The Hebrew version was based on the same translated questionnaire previously used in an Israeli population, and has shown good psychometric properties [40]. The internal consistency of the scale in the current study was high (Cronbach's α = 0.94).

The anxiety and depression domains of the Patient Reported Outcomes Measurement Information System (PROMIS; [41]) were used in order to assess the severity of depression and anxiety symptoms. Each domain consists of eight items that assess common symptoms of anxiety or depression over the past week. Ratings are made on a 5-item Likert-type response scale, ranging from 1 (= *not at all*) to 5 (= *very much*). Scores were calculated according to the PROMIS scoring manuals, in which raw summed scores are converted into standardized T-scores (*M* = 50, *SD* = 10) based on the U.S. general population reference sample, with higher scores indicating greater symptom severity [42]. A previous study provided support for the reliability and construct validity of the PROMIS anxiety and depression measures in a cancer population [43]. In the current study, we used a validated Hebrew version of the questionnaire [44]. The internal consistency of the scale in the current study was high (Cronbach's α = 0.91 for anxiety and 0.93 for depression).

### Statistical analysis

Descriptive statistics and bivariate correlations were computed for study variables. Reliability analyses, as well as all statistical procedures, were conducted using IBM SPSS Statistics (version 31). Next, a regression analysis was conducted using independent variables SSA, DERS, and the interaction term SSA × DERS, with FCR scores as the dependent variable. Age, gender, duration since treatment completion (in months), and anxiety symptom severity were included as control variables based on previous empirical evidence and current bivariate correlations with FCR [4, 6, 45].

Symptoms of anxiety and depression were highly correlated; therefore, only one indicator of general psychological distress was included in the regression analyses to reduce redundancy and potential multicollinearity. Anxiety symptom severity was selected given its well-established association with FCR [45] and its stronger correlation with FCR in the current sample.

Prior to regression analysis, the main predictor and outcome variables were standardized (z-scores) to facilitate interpretation. A multicollinearity test was performed for all predictors. The test revealed good tolerance values (close to 1) and variance inflation factor (VIF) values, indicating no multicollinearity among the predictors. The control variables were entered at step 1 (model 1), SSA and DERS were entered at step 2 (model 2), and the interaction term SSA x DERS was entered at step 3 (model 3). Following a significant interaction effect, simple slopes analyses were conducted using the PROCESS macro for SPSS (Version 4.2; [46]). Conditional effects were initially examined at low (− 1 SD), mean, and high (+ 1 SD) levels of DERS for descriptive visualization purposes. In addition, Johnson–Neyman analyses were conducted to identify the specific range of DERS values at which the association between SSA and FCR transitioned between statistical significance and non-significance across the continuous moderator.

## Results

### Descriptive statistics of the study variables

Demographic and medical characteristics of the sample are presented in Table 1. The mean FCR score was 19.63 (*SD* = 6.90), and did not differ by gender, *t*(114) = 0.65, *p* = 0.52; with females (*M* = 19.38, *SD* = 6.85) and males (*M* = 20.33, *SD* = 7.14) reporting comparable levels. Similarly, the mean SSA score was 2.80 (*SD* = 0.70), and did not differ by gender, *t*(114) = −0.88, *p* = 0.38, with females (*M* = 2.84, *SD* = 0.74) and males (*M* = 2.71, *SD* = 0.54) showing similar scores. The mean DERS score was 82.36 (*SD* = 23.51), again with no significant gender differences, *t*(114) = 0.55, *p* = 0.58; with females (*M* = 81.65, *SD* = 23.03) and males (*M* = 84.40, *SD* = 25.12) reporting comparable levels.

**Table 1:** Demographic and Medical Characteristics of Participants (N = 116)

Variable	Mean	*SD*	Range
Age	47.24	13.23	20–72
Years of education	15.28	3.06	9–26
Time since diagnosis (months)	37.15	26.36	4–116
Time since last treatment (months)	26.29	23.86	1–96
Variable	Category	N	%
Sex	Male	30	26
Marital status	Married	67	58
	Divorced	24	20
	Single	23	20
	Widowed	2	2
Religiosity	Secular	65	56
	Traditional	29	25
	Religious	22	19
Cancer type	Breast	51	44
	Lymphoma	23	20
	Gastrointestinal	22	19
	Others	20	17
Cancer stage	0	3	3
	I	12	10
	II	44	38
	III	35	30
	IV	22	19
Cancer treatment completed	Chemotherapy	95	82
	Surgery	79	68
	Radiation therapy	72	62
	Biological therapy	34	29
	Hormone therapy	16	14
Recurrence	Yes	9	8

As shown in Table 2, bivariate correlations indicated that higher levels of FCR were significantly associated with younger age and shorter time since treatment completion (*r* = –0.23, *p* < 0.05; *r* = –0.24, *p* < 0.01; respectively). SSA and DERS scores were significantly associated with younger age (*r* = −0.26, *p* < 0.01; *r* = −0.30, *p* < 0.01; respectively). Significant positive associations were observed between SSA and FCR (*r* = 0.29, *p* < 0.01), between DERS and FCR (*r* = 0.37, *p* < 0.001), and between SSA and DERS (*r* = 0.28, *p* < 0.01). Depression and anxiety symptom severity were positively correlated with SSA, DERS, and FCR (*p*'s < 0.05, see Table 2).

**Table 2:** Bivariate Correlations Among Primary Study Variables (N = 116)

	**1**	**2**	**3**	**4**	**5**	**6**	**7**
1. SSA	—						
2. DERS	0.28**	—					
3. FCR	0.29**	0.37***	—				
4. Depression	0.22*	0.63***	0.28**	—			
5. Anxiety	0.26**	0.59***	0.46***	0.70***	—		
6. Age	−0.26**	−0.30**	−0.23*	−0.22*	−0.17	—	
7. Sex	0.08	−0.05	−0.06	0.03	0.02	.13	—
8. Time (months)	−0.07	−0.08	−0.24**	−0.10	−0.12	0.37***	−0.06

SSA = somatosensory amplification; DERS = difficulties in emotion regulation; FCR = fear of cancer recurrence; Age = years; Sex = high value represents females; Time = time since treatment completion

^*^ *p* <.05, ** *p* <.01, *** *p* <.001

### Test of hypotheses

As summarized in Table 3, model 1 included age, gender, time since treatment completion, and anxiety symptoms severity^1^ as control variables and was statistically significant, *F*(4, 111) = 9.60, *p* < 0.001, explaining 25.7% of the variance in FCR. Model 2 added SSA and DERS as predictors. Although the overall model remained statistically significant, *F*(6, 109) = 7.39, *p* < 0.001, the addition of SSA and DERS did not significantly improve the model fit (Δ*R*^2^ = 0.032, *p* = 0.09). In this model, neither SSA (*β* = 0.17, 95% CI [−0.01, 0.34], *p* = 0.06) nor DERS (*β* = 0.10, 95% CI [−0.11, 0.31], *p* = 0.35) significantly predicted FCR.

**Table 3:** Hierarchical Regression Models Predicting FCR

Predictor	Model 1 β	Model 2 β	Model 3 β
Age	−0.09	−0.02	−0.16
Gender	−0.07	0.08	−0.09
Time (months)	−0.16	−0.18*	−0.18*
Anxiety	0.42***	0.33**	0.31**
SSA	—	.17	.16
DERS	—	.10	.13
SSA × DERS	—	—	−.18*
R^2^	.26	.29	.32
ΔR^2^	—	.03	.03*
F for model	9.60***	7.39***	7.27***
Df	(4, 111)	(6, 109)	(7, 108)

β = standardized regression coefficient. SSA = somatosensory amplification; DERS = difficulties in emotion regulation scale; Time = time since treatment completion

^*^p <.05. **p <.01. ***p <.001

Model 3 introduced the interaction term between SSA and DERS, which significantly improved the model, Δ*R*^2^ = 0.031, *F*_change_(1, 108) = 4.96, *p* = 0.028. The full model was statistically significant, *F*(7, 108) = 7.27, *p* < 0.01, explaining 32.0% of the variance in FCR. The interaction term was statistically significant (*β* = –0.18, 95% CI [−0.31, −0.02], *p* < 0.03), indicating that the association between SSA and FCR depended on levels of ER difficulties.

Johnson–Neyman analyses further indicated that SSA was significantly associated with FCR only at levels of ER difficulties below approximately average levels (z =  − 0.08), whereas the association became non-significant at higher levels of ER difficulties.

**Fig. 1:**
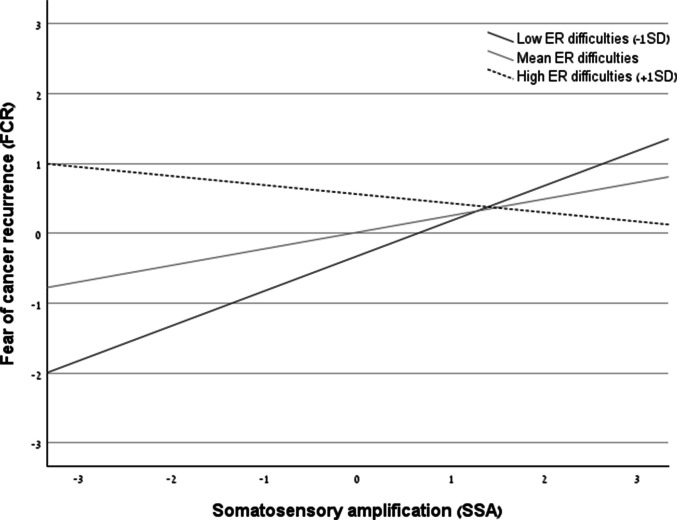
Association between somatosensory amplification (SSA) and fear of cancer recurrence (FCR) at low (− 1 SD), mean, and high (+ 1 SD) levels of emotion regulation difficulties (DERS). Solid lines represent statistically significant simple slopes; the dashed line represents a non-significant simple slope

As shown in Fig. 1, SSA was not significantly associated with FCR at high levels of ER difficulties (+ 1 SD; *b* = −0.03, *SE* = 0.12, *p* = 0.79), but was positively associated at mean (*b* = 0.19, *SE* = 0.09, *p* < 0.03) and low levels (–1 SD; *b* = 0.32, *SE* = 0.11, *p* < 0.01). This pattern suggests that the relationship between SSA and FCR was evident at low and mean levels of ER difficulties, but not at high levels.

## Discussion

This study examined the associations between SSA, ER difficulties, and FCR among cancer survivors. In bivariate analyses, both SSA and ER difficulties were positively associated with FCR. However, after controlling for age, gender, time since treatment completion, and anxiety symptom severity, the main effects of SSA and ER difficulties were no longer statistically significant, whereas the interaction between SSA and ER difficulties was statistically significant. Specifically, the positive association between SSA and FCR was evident at low and mean levels of ER difficulties, but not at high levels. These findings suggest that the relationship between heightened bodily sensitivity and FCR may differ according to levels of ER difficulties and highlight the complex interplay between bodily sensitivity, ER processes, and general psychological distress in cancer survivorship.

Although SSA has been widely examined across medical and psychiatric conditions, including hypochondriasis, fibromyalgia, and anxiety disorders [e.g., 14, 47], it has received limited attention in the context of post-treatment cancer survivorship, where bodily sensations occur against a background of uncertainty and perceived threat of recurrence [15, 16]. Examining SSA in relation to FCR, therefore, extends research on this construct to a distinct survivorship context.

In bivariate analyses, SSA was positively associated with FCR, supporting theoretical models that emphasize heightened attention to bodily sensations in recurrence fears [9]. Prior research has frequently examined symptoms such as pain or fatigue as correlates of FCR [e.g., 12]. Our findings extend this literature by highlighting the potential role of subjective interpretations and amplification of bodily sensations, beyond symptom presence alone. Specifically, FCR may relate not only to the experience of physical symptoms but also to the tendency to perceive such sensations as intense, distressing, or threatening, which are core characteristics of SSA. However, after controlling for demographic variables and anxiety symptom severity, SSA was no longer independently associated with FCR, suggesting that the association between SSA and FCR overlaps with the variance accounted for by these variables. While SSA and FCR may share processes such as hypervigilance and catastrophizing [48], further work is needed to distinguish trait-like tendencies toward bodily amplification from recurrence-specific interpretations of bodily sensations.

ER difficulties have been robustly linked to a wide range of psychiatric conditions, including anxiety disorders, eating disorders, depression, posttraumatic stress disorder, and substance use disorders [26–28]. In the present study, ER difficulties were positively associated with FCR at the bivariate level, consistent with prior literature linking ER-related processes to health anxiety and illness-related distress [29]. However, after controlling for demographic variables and anxiety symptom severity, ER difficulties were no longer independently associated with FCR, suggesting substantial overlap between ER difficulties and distress-related processes.

A growing body of research has examined associations between specific ER strategies (e.g., suppression, rumination, avoidance) and FCR, predominantly among patients undergoing active cancer treatment [33, 49–53]. In contrast, substantially less research has focused on broader ER difficulties among cancer survivors [30, 34, 54], a population that differs from patients in active treatment in terms of ongoing uncertainty, perceived vulnerability, and long-term self-regulatory demands [55, 56]. Moreover, ER difficulties, as assessed by the DERS [25], have been examined only rarely in relation to FCR [2].

Although ER difficulties did not independently predict FCR in the adjusted model, the significant interaction between ER difficulties and SSA suggests that bodily amplification processes may become relevant to FCR under specific levels of ER difficulties. These findings point to a potentially more conditional role of ER difficulties in survivorship-related distress, whereby ER difficulties may shape how bodily sensations are interpreted or responded to rather than directly contributing to FCR in a uniform manner. Importantly, contrary to our hypothesis that at higher levels of ER difficulties, the association between SSA and FCR would be stronger, the positive association between SSA and FCR was observed only among survivors with low and mean levels of ER difficulties. In contrast, among survivors with high levels of ER difficulties, the association between SSA and FCR was not statistically significant. Johnson–Neyman analyses further indicated that SSA was significantly associated with FCR only at lower levels of ER difficulties, whereas the association became non-significant as ER difficulties approached and exceeded average levels. These findings suggest that bodily amplification may be linked to recurrence fears primarily under conditions of relatively lower ER difficulties.

The finding that the association between SSA and FCR was evident primarily at lower levels of ER difficulties may suggest that heightened attention to and interpretation of bodily sensations becomes more closely linked to recurrence fears under conditions of relatively lower ER difficulties. One possible explanation is that individuals reporting lower ER difficulties may be more attentive to, aware of, or able to differentiate internal bodily experiences, thereby increasing the likelihood that amplified bodily sensations become associated with recurrence-related concerns. This interpretation is broadly consistent with findings by Harel et al. [34], who reported that inflammatory cytokines were more strongly associated with depressive symptoms among breast cancer survivors reporting higher emotional awareness, a construct conceptually related to attention to and differentiation of internal emotional states. Harel et al. [34] further proposed that heightened emotional awareness may reflect increased sensitivity to interoceptive cues, consistent with theoretical models linking emotional awareness and interoception [57]. Taken together, these findings raise the possibility that lower ER difficulties do not necessarily reduce sensitivity to bodily experiences, but may instead be associated with greater awareness or processing of internal sensations, potentially strengthening the association between bodily amplification and recurrence-related fears under some conditions.

Several alternative interpretations of the present findings should also be considered. First, the bivariate association between SSA and FCR may partly reflect broader distress-related processes, particularly given that SSA was no longer independently associated with FCR after controlling for anxiety symptom severity and demographic variables. From this perspective, heightened distress or hyperarousal may contribute to both increased recurrence fears and heightened attention to bodily sensations, resulting in overlap between these constructs. In addition, the cross-sectional design precludes conclusions regarding directionality or temporal sequencing. Elevated FCR may increase the monitoring and interpretation of bodily sensations, thereby contributing to higher SSA under certain psychological conditions. More broadly, the present findings suggest that the association between bodily amplification and FCR may not be uniform across survivors but may instead depend on individual differences in ER-related processes. Longitudinal and experimental studies are needed to clarify reciprocal relationships and temporal dynamics among SSA, ER difficulties, and FCR.

### Limitations

Several limitations should be noted. First, the medically heterogeneous sample, spanning various cancer types, stages, and treatments, enhances generalizability but may obscure cancer-specific patterns. Second, the sample was predominantly female (74%), with 44% being breast cancer survivors, potentially influencing results given gender differences in FCR [58]. This imbalance likely reflects recruitment bias, as women are more active in online research [59]. Third, reliance on self-report measures introduces potential biases, such as social desirability and recall errors; future studies should incorporate behavioral, clinician-rated, or physiological assessments. Fourth, the cross-sectional design limits causal inference; longitudinal studies are needed to clarify temporal and bidirectional relationships among SSA, ER difficulties, and FCR. Lastly, while we controlled for some covariates (age, gender, time since treatment completion, and anxiety symptom severity), unmeasured factors like psychiatric history, pain, and comorbidities may have influenced the findings.

### Clinical implications

The present findings suggest that ER-related processes may play a complex role in the relationship between bodily amplification and FCR during survivorship. Specifically, the association between SSA and FCR was evident primarily at lower-to-average levels of ER difficulties, indicating that heightened bodily sensitivity may become linked to recurrence fears under some regulatory conditions but not others. Although interventions that strengthen ER abilities are generally assumed to improve emotional well-being among cancer survivors [60], the present findings suggest that the relationship between ER-related processes and FCR may be more complex. In some cases, certain aspects of ER-related functioning, such as increased attention to emotional and bodily experiences, may heighten awareness of somatic sensations and thereby strengthen their association with recurrence fears. Accordingly, future longitudinal and intervention-based research is needed to clarify how changes in ER-related functioning influence the relationship between bodily amplification and FCR over time.

In addition, the observed association between SSA and FCR among survivors with low and mean levels of ER difficulties suggests that attention to how bodily sensations are perceived and interpreted may be particularly relevant for this subgroup. Approaches targeting bodily vigilance and the interpretation of bodily sensations, including mindfulness-based and cognitive-behavioral interventions, may be relevant for FCR [61–63]. In particular, mindfulness-based cognitive therapy has shown promise in reducing FCR and related distress among cancer survivors [62, 63]. Overall, tailoring psychosocial support based on individual profiles of ER and bodily awareness may be a promising direction for future research aimed at improving the personalization of survivorship care.

## Conclusions

In sum, the present study examined associations among SSA, ER difficulties, and FCR in cancer survivors. Although SSA and ER difficulties were positively associated with FCR at the bivariate level, these associations were no longer independently significant after accounting for demographic variables and anxiety symptom severity. Importantly, the association between SSA and FCR differed according to levels of ER difficulties, such that SSA was significantly associated with FCR at low and mean levels of ER difficulties, but not at high levels. These findings suggest that heightened bodily sensitivity may become linked to recurrence fears primarily under conditions of relatively lower ER difficulties. More broadly, the findings underscore the importance of considering how bodily amplification processes interact with emotional functioning when understanding FCR in survivorship. Importantly, the post-treatment survivorship period is often characterized by ongoing uncertainty, heightened attention to bodily sensations, and greater reliance on self-monitoring following the end of active treatment [55, 56], making this a period of heightened psychological vulnerability for many survivors.

## Data Availability

Data will be available upon request from the corresponding author.

## Footnotes

1 In unadjusted models, both SSA and DERS were significantly associated with FCR, and the SSA × DERS interaction was significant. After adjustment for age, gender, time since treatment completion, and anxiety symptom severity, the interaction term remained significant, whereas the main effects of SSA and DERS were attenuated and no longer statistically significant.
